# Retraction Note: Molecular mechanisms in progression of HPV-associated cervical carcinogenesis

**DOI:** 10.1186/s12929-019-0545-6

**Published:** 2019-07-04

**Authors:** Sadhana M. Gupta, Jayanti Mania-Pramanik

**Affiliations:** Department of Infectious Diseases Biology, National Institute for Research in Reproductive Health, J.M. Street, Parel, Mumbai, 400012 India

**Retraction to Journal of Biomedical Science (2019) 26:28**

**https://doi.org/10.1186/s12929-019-0520-2**

The Editor-in-Chief has retracted this article [1] due to significant overlap with previously published articles [2–5]. Both authors agree with this retraction.
